# Polyparasitism and Anaemia Among Women of Reproductive Age in Kilifi County—Kenya

**DOI:** 10.1155/jotm/2791451

**Published:** 2026-02-11

**Authors:** Matano Mjomba, Simon Muriu, Victor Tunje Jeza

**Affiliations:** ^1^ Department of Biological Sciences, Pwani University, Kilifi, Kenya, pu.ac.ke; ^2^ Department of Medical Sciences, Technical University of Mombasa, Mombasa, Kenya, tum.ac.ke

**Keywords:** anaemia, Kilifi County, polyparasitic infections, single infections, WRA

## Abstract

**Background:**

Polyparasitism is commonly associated with *Plasmodium* species*, Schistosoma* species and soil‐transmitted helminths. Anaemia results from a variety of causes, including parasitic infections. Women of reproductive age (WRA) and children below the age of five are disproportionately affected by polyparasitic infections, putting them at risk of anaemia. The current study was conducted to evaluate the association of polyparasitic infections with anaemia among WRA in Kilifi.

**Materials and Methods:**

A cross‐sectional study was conducted among 478 WRA in Rabai and Magarini subcounties in Kilifi County. Blood samples were collected and analysed for *Plasmodium falciparum*, determination of haemoglobin (HB), and classification of anaemia, while urine and stool samples were tested for *Schistosoma haematobium* and soil‐transmitted helminths, respectively. Data were analysed using R software, and the overall magnitude of parasitic coinfections, anaemia and their associated factors was determined by chi‐square and t‐test. The differences were considered statistically significant if *p*‐value was ≤ 0.05. The means of HB were determined to evaluate the synergistic effect of different single parasites in polyparasitism on HB at a 95% confidence interval.

**Results:**

The overall prevalence of parasitic infections was 26.9%. Polyparasitism had a prevalence of 1.8% due to concurrent infection of *P. falciparum* and *S. haematobium, S. haematobium* and hookworm, *S. haematobium,* hookworm and *Ascaris lumbricoides,* whose prevalence was 0.8% (*p* < 0.001). 0.8% (*p* < 0.001) and 0.2% (*p* < 0.001) *respec*tively. The prevalence of anaemia was 16.5%, while the prevalence of normocytic and microcytic anaemia was 63% and 37%, respectively. There was no synergy between different parasites in polyparasitism and HB.

**Conclusion:**

The study findings indicated that the prevalence of polyparasitism was low. Polyparasitic infections involving *S. haematobium* and *P. falciparum* were most common in the region. Anaemia was common among *P. falciparum* and *Trichuris trichiura-*infected participants, while normocytic and microcytic anaemia were common in both infected and noninfected women.

## 1. Introduction

Polyparasitic infection is the concurrent infection of a single host with multiple parasitic species [1]. Polyparasitic infections are prevalent in developing countries where basic resources such as clean water, proper housing, proper planned water management and sewage systems are not readily available [2]. *Plasmodium, Schistosoma* and soil‐ttransmitted helminths (STHs), that is, roundworms (*Ascaris lumbricoides*), whipworm (*Trichuris trichiura*) and hookworm (*Necator americanus* and *Ancylostoma duodenale*), are the most common parasites found in concurrent infections [3]. The epidemiologic coexistence of these parasites, particularly *Plasmodium* and helminths, has frequently been observed in Africa, and by 2017, 451 million people were at risk of being infected with hookworm, 435 million with *T. trichiura*, 190 million with schistosomiasis and 800 million with *A. lumbricoides* [1].

Apart from the considerable spatial and epidemiologic distribution, these parasites may exhibit great morphological diversity, which increases the chance of polyparasitic infections among single susceptible hosts [4]. Polyparasitic infections in humans may be antagonistic or synergistic with varying impacts on human health [5]. Apart from being ubiquitous in tropical regions, *Plasmodium falciparum, Schistosoma haematobium* and STH infestations are major causes of anaemia and are thus a major cause of common morbidities in tropical countries [6]. Women and children are the most vulnerable to polyparasitic infections because children habitually play and come into contact with faecally contaminated soils, which can be a source of STH transmission. Further, they have underdeveloped immunity and may be exposed to parasitic vectors like mosquitoes when they sleep without mosquito nets. Women can easily be infected with parasitic infections because most of their daytime is spent performing household roles and fetching firewood from the forests and water from ponds, dams and rivers for their domestic use [7].

Most studies done in Kenya established the endemicity of intestinal parasites, malaria and schistosomiasis, with a few having looked at polyparasitic infections and their association with haemoglobin (HB) and the vulnerability to anaemia resulting from coinfections [8]. Therefore, a need for understanding the association between polyparasitic infections and anaemia and a paucity of data on this issue informed the need for this study.

## 2. Materials and methods

### 2.1. Research Design Settings

**Figure 1:**
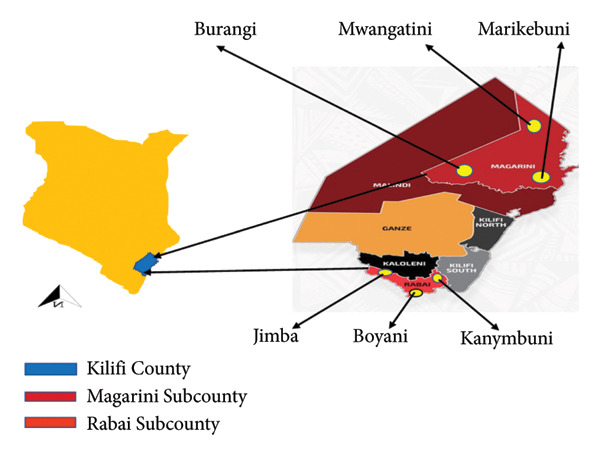
A map of Rabai and Magarini subcounties.

A cross‐sectional study design was adopted in two purposive sampled subcounties of Kilifi County, namely, Rabai and Magarini subcounties (Figure 1). Rabai and Magarini subcounties were selected because of the prevailing parasitic infection endemicity.

Rabai Sub‐County lies between latitudes 3° 38′ and 3° 59′ south and longitudes 39° 21′ and 39° 39′ east and has a population size of approximately 120,813 people [9]. Magarini Sub‐County lies between 3° 2′ 0″ south and 40° 4′ 0″ east and has a population size of approximately 191,160 people [10].

Kilifi County has a distinct hot and humid climate with bimodal rainfall averaging at 102.11 mm and a temperature range of 27°C to 28°C. About 70% of the inhabitants are low‐income earners whose main water sources are dams, rivers, shallow wells, springs and boreholes. Most villagers live in extended families whose hygienic standards are compromised by poverty [11]. These and other factors modify the environment and increase the risk of parasitic transmission [4].

### 2.2. Target Population/Sample Size/Sampling Technique

The study participants were 478 women aged between 15 and 49 years randomly selected from the study area. Three villages in each of the two subcounties were selected. These were Marikebuni, Mwangatini and Boyani in Magarini subcounty and Kanyumbuni, Boyani and Jimba in Rabai subcounty (Figure 1). Venous blood was collected from the study participants through venipuncture by a qualified phlebotomist. For urine samples, the study participants were issued with a plastic screw‐capped container and instructed to collect a mid‐morning sample of urine between 10:00 am and 2:00 pm. A stool container was also issued to the participant for stool collection. All samples were appropriately labelled and transported in a cooler box to the designated testing laboratory.

### 2.3. Laboratory methods

Blood was tested for *P. falciparum* and HB concentration. *P. falciparum* was tested by a rapid diagnostic antigen test kit (ICT Diagnostics, Australia) and microscopy. The rapid diagnostic technique was primarily used for initial diagnosis to allow treatment of positive participants in the field. In microscopy, 10% Giemsa‐stained thick and thin blood smears were microscopically examined at × 100 magnification. For thick smears, one hundred microscopic fields were examined before concluding that a smear was negative for *P. falciparum*. For accuracy, all blood smears were examined twice by microscopy. To determine the parasite density per microlitre of blood, the following formula was applied: (Number of trophozoites × 8000)/Number of leucocytes. *P. falciparum* infection intensity was classified into light, moderate, heavy, or very heavy infections when the parasite count was 1–499 parasites/mL, 500–1999 parasites/mL, 2000–9999 parasites/mL, and 10,000 parasites/mL, respectively.

HB concentration was tested on a haematology analyzer (Nihon Kohden Celltac MEK8222). Anaemia was defined into mild, moderate and severe based on HB concentrations of 10.0 g/d, between 8.0 and 10.0 g/d and between 6.5 and 7.9 g/dL respectively and also classified into three categories, that is, normocytic, microcytic and macrocytic based on MCV.

Urine samples were tested for *S. haematobium* eggs by nuclear pore urine filtration technique. After filtration, the filter was immediately microscopically examined at × 40 magnification. Any number of *S. haematobium* eggs greater than zero found in 10 mL of urine confirmed the presence of *S. haematobium* infection.

The quantitative Kato–Katz technique for microscopic detection of eggs in stool was used to examine duplicate stool samples for the presence of STHs. The stool was processed, and final specimens were microscopically examined. To ascertain the intensity of infection, the number of eggs counted was multiplied by a factor of 50 and classified as light, moderate and heavy infections as per the WHO guidelines [12].

### 2.4. Data Analysis

Data were analysed in R software (Version 3.6.1). We treated age as a categorical variable and stratified it into 5‐year groups. Participants were considered infected with *P. falciparum* and *S. haematobium* when tested positive for either thick or thin Giemsa‐stained blood smears and the nucleopore filtration method, respectively. Prevalence of *P. falciparum* and *S. haematobium* were determined by proportions at a 95% confidence interval. Statistical significance of the differences was considered if *p*‐value was < 0.05.

### 2.5. Ethical Consideration

The study protocol was reviewed by the Scientific and Ethical Review Committee of the Kenya Medical Research Institute (KEMRI) (SERU # KEMRI/SERU/ESACIPAC/3684). Licensing of the study was acquired from the Kenya National Commission for Science, Technology, and Innovation (NACOSTI) in accordance with the rules and regulations that govern all studies in Kenya. Permission to carry out the study in Kilifi County was sought through the County Department of Health services. All participants provided a signed consent in Swahili or English after the study was explained. For those below 18 years, the legal guardians or parents signed the consent form on their behalf after assent. For women of reproductive age (WRA) who may not have been able to read for any reason, the consent form was read to them and signed by imprinting using the thumb finger. All participants were made aware that they could withdraw from the study at any time.

## 3. Results

### 3.1. Prevalence of *Parasitic Infections* Among WRA in Kilifi County

**Figure 2:**
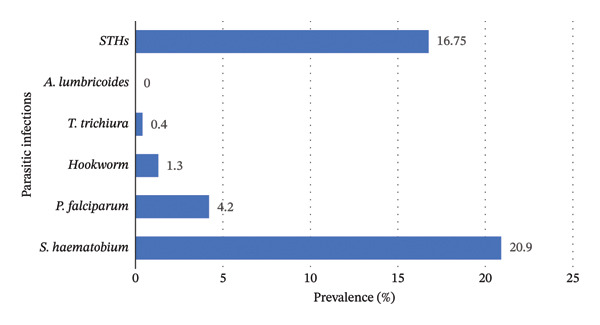
Prevalence of parasitic infections among WRA in Kilifi County.

Five parasite species, namely *S. haematobium, P. falciparum,* hookworm, *A. lumbricoides* and *T. trichiura,* were encountered in this study (Table 1). *S. haematobium* (20.9%) (*p* < 0.001) was the predominant parasite detected in the region, followed by *P. falciparum* (4.2%) (*p* < 0.001) and hookworm (1.3%) (*p* < 0.001). 71.3% of the study population were noninfected (Figure 2).

**Table 1:** Distribution of parasitic infections among WRA by village.

Infection	Women by village. No. (%)						
	Magarini Sub‐County			Rabai Sub‐County			
	Marikebuni *n* = 39	Mwangatini *n* = 95	Burangi *n* = 57	Kanyumbuni *n* = 86	Boyani *n* = 108	Jimba *n* = 93	*p* value^∗^
*Plasmodium falciparum*	2 (5.1)	16 (16.8)	0 (0)	5 (5.8)	1 (0.9)	0 (0)	< 0.001
*Schistosoma haematobium*	0 (0)	13 (13.7)	17 (29.8)	9 (10.5)	27 (25)	34 (36.6)	< 0.001
Hookworm	0 (0)	1 (1.5)	0 (0)	1 (1.2)	3 (2.8)	3 (3.2)	0.221
*Ascaris lumbrcoides*	0 (0)	0 (0)	0 (0)	0 (0)	0 (0)	0 (0)	< 0.001
*Trichuris trichiura*	0 (0)	0 (0)	0 (0)	1 (1.2)	0 (0)	1 (1.1)	0.549

^∗^Significance at *p* < 0.05.

### 3.2. Distribution of Parasitic Infections Among WRA in Kilifi County

*S. haematobium* and *P. falciparum* were the most predominant parasites. *S. haematobium* was found in Jimba (36.6%), Burangi (29.8%), Boyani (25%), Mwangatini (13.7%) and Kanyumbuni (10.5%), while *P. falciparum* infections were recorded in Mwangatini (16.8%), Kanyumbuni (5.8%), Marikebuni (5.1%) and Boyani (0.9%) villages. Hookworm infections (*p* = 0.221) were found in all villages of Rabai Sub‐County and only Mwangatini village in Magarini Sub‐County (Table 1).

### 3.3. Distribution of Parasitic Infections by Age

Infections with all the identified parasites declined with an increase in age, though not significant (*p* = 0.9985). The study showed that 34 (7.1%) and 10 (2.1%) WRA of the age between 15 and 21 were infected with *S. haematobium* and *P. falciparum,* respectively. Hookworm infections were mostly recorded among the middle‐aged women between the ages of 22–28 years and 29–35 years (Table 2).

**Table 2:** Prevalence of parasitic infections in relation to age.

Variables	Parasitic infections. no (%)								
Age category	Monoparasitism					Polyparasitism			*p* value^∗^
	Pf	Sh	Hw	Al	Tt	Pf + Sh	Sh + Hw	Sh + H + Al	
15–21	10 (2.1)	34 (7.1)	0 (0)	0 (0)	1 (0.2)	3 (0.6)	2 (0.4)	1 (0.2)	0.999
22–28	3 (0.6)	31 (6.5)	3 (0.6)	0 (0)	0 (0)	1 (0.2)	2 (0.4)	0 (0)	
29–35	4 (0.8)	14 (2.9)	2 (0.4)	0 (0)	1 (0.2)	0 (0)	0 (0)	0 (0)	
36–42	2 (0.4)	12 (2.5)	1 (0.2)	0 (0)	0 (0)	0 (0)	0 (0)	0 (0)	
43–49	1 (0,2)	9 (1.9)	0 (0)	0 (0)	0 (0)	0 (0)	0 (0)	0 (0)	

Total	20 (4.1)	100 (20.9)	6 (1.2)	0 (0)	2 (0.4)	4 (0.8)	4 (0.8)	1 (0.2)	
	128 (26.8)					9 (1.8)			

*Note:* Hw: hookworm.

Abbreviations: Al, *A. lumbricoides*; Pf, *P. falciparum*; Sh, *S. haematobium*.

^∗^Significance at *p* < 0.05.

### 3.4. Prevalence of Polyparasitic Infections

The overall prevalence of polyparasitic infections was 1.8%; 1.6% for double and 0.2% for triple infections. Double infections resulted from concurrent infections of *P. falciparum* and *S. haematobium* (0.8%) and *S. haematobium* and hookworm (0.8%) while triple infection consisted of *S. haematobium*, hookworm and *A. lumbricoides* (0.2%). *S. haematobium* dominated in all forms of polyparasitism. Polyparasitism was more common among women between the ages of 15 and 28 years (Table 2).

### 3.5. Prevalence of Anaemia Among Women in Kilifi County

**Figure 3:**
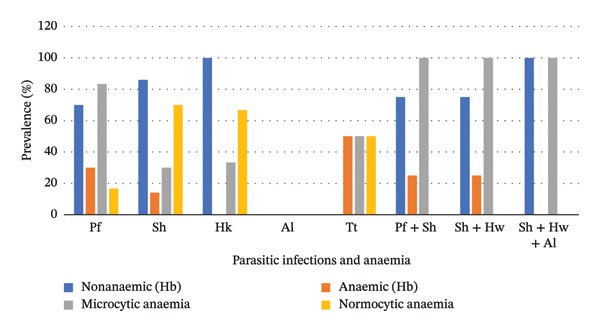
Association between anaemia and parasitic infections. Key: Pf, *P. falciparum*; Sh, *S. haematobium*; Hw, hookworm; Al, *A. lumbricoides*; Tt, *T. trichiura*.

Anaemia was classified based on HB concentration and MCV. On the basis of HB concentration, those WRA with an HB concentration of less than 11 g/dL were considered anaemic, while those with an HB concentration above 11 g/dL were considered healthy. On the basis of MCV, anaemia was classified into microcytic (MCV of less than 77 fL), normocytic (MCV of between 77 and 99 fL) and macrocytic (MCV of greater than 99 fL). Overall, 79 (10.56%) tested positive for anaemia. Of these, 55 (69.62%) and 24 (30.38%) had microcytic and normocytic anaemia, respectively (Figure 3).

The overall prevalence of anaemia was 10.56%. 4.4% of the anaemic cases were infected by single parasites (*p* < 0.001) while 0.2% of WRA had polyparasitism of *P. falciparum* and *S. haematobium* (*p* = 0.317) while coinfection of *S. haematobium* and hookworm accounted for 0.2% (*p* value 0.317) of the infections (Figure 3).

Microcytic and normocytic anaemia were encountered at a prevalence of 69.62% and 30.38%, respectively. Approximately 32.3% WRA had microcytic anaemia with a single infection of either *P. falciparum, S. haematobium,* hookworm*, A. lumbricoides or T. trichiura* (*p* < 0.001). A positive association between polyparasitism and anaemia was recorded (*p* = 0.046): Polyparasitic infected individuals had microcytic anaemia with either polyparasitism of *P. falciparum* and *S. haematobium* (0.8%) (*p* value 0.046), *S. haematobium* and hookworm (0.8%) (*p* = 0.046) or *S. haematobium,* hookworm and *A. lumbricoides* (0.2%) (*p* value 0.317) (Figure 3).

### 3.6. Synergistic Effect of *P. falciparum*, *S. haematobium*, Hookworm, *T. trichiura* and *A. lumbricoides* on HB

The mean HB of noninfected WRA was 12.28 g/dL (95% CI 12.12–12.44). Therefore, no parasitic infection contributed to reduced anaemia. Hookworm‐infected WRA had an HB mean of 13.0 g/dL (95% CI 12.16–13.99), while *P. falciparum* and *T. trichiura* infected WRA had HB means of 11.2 g/dL (95% CI 10.30–12.11) and 11.86 g/dL (95% CI 2.57–25.89), respectively. All cases of polyparasitism recorded a mean HB of above 12.1 g/dL (95% CI 9.68​ *A. lumbricoides* (Table 3).

**Table 3:** The mean values of haemoglobin among WRA in Kilifi County.

Variables	*N*	Mean haemoglobin g/dL	95% CI
None infected	341	12.28	12.12–12.44

*Mono parasitism*			
Pf	20	11.20	10.30–12.11
Sh	100	12.47	12.17–12.78
Hw	6	13.0	12.16–13.99
Tt	2	11.86	−2.57–25.89

*Polyparasitism*			
Pf + Sh	4	12.13	9.68–14.59
Sh + Hw	4	12.78	9.65–15.91
Sh + Hw + Al	1	12.54	—

## 4. Discussion

The present study examined the burden of polyparasitism among WRA in Kilifi County. While most studies have been separately done on malaria and STHs in children, to our knowledge, this was the first study done to establish the relationship of polyparasitism and anaemia among WRA in Kilifi.

*P. falciparum* recorded a prevalence of 4.2%, which was low compared to a prevalence of between 9% and 18% in a study done in 2020 among pregnant women in Kwale County [13]. These findings might be as a result of the immense government malaria intervention measures through the 2009–2017 National Malaria Strategy (NMS), which recommended the provision of intermittent preventive treatment for malaria in pregnancy with sulfadoxine–pyrimethamine during antenatal care visits, prompt diagnosis and effective treatment of malaria infections, use of long‐lasting insecticidal nets in all areas of moderate to high malaria transmission, and building the capacity of healthcare providers and strengthening the supply chain to deliver diagnostic tests and quality‐assured medicines [14].

*S. haematobium* was the most prevalent parasite (20.1%). Villages in Rabai Sub‐County were the mostly affected compared to Magarini Sub‐County. The variance in the prevalence of *S. haematobium* may be attributed to the fact that the most common water sources in Rabai Sub‐County are rivers, dams, ponds and wells, while some parts of Magarini, like Marikebuni, obtain their water from taps, while the remaining two villages use stored rainwater and river water [15]. In a similar study done in Kwale County [16], the prevalence of *S. haematobium* was reported to be 3.8%, which is lower than the current study findings. The upsurge of *S. haematobium* in the study area may be as a result of lack of clean water supply, despite the fact that programmes like the National School‐based Deworming Programmes that were successful in Kwale were also used in the area [17]. This therefore means that for such interventions to be successful, a clean water supply should be provided to reduce the reinfection due to contact of women and parasites when they visit rivers and ponds to fetch water.

The overall prevalence of STHs was 16.75%, which indicates a downward trend in the area; a previous study on the status of STHs among pregnant women attending antenatal clinics at Kilifi County Hospital recorded a prevalence of 22.2% [13]. Government parasitic infection control initiatives targeting school‐age children that were conducted between 2012 and 2017 [17], the annual and biannual distribution of albendazole to schools through the TUMIKIA project between 2015 and 2017 [18] and the provision of clean water supplies, health education and use of toilets might have resulted in the drastic reduction of STH transmission and infection in the area [11].

The current study also recorded a 1.8% prevalence of polyparasitic infections among WRA with *P. falciparum* and *S. haematobium* (0.8%), *S. haematobium* and hookworm (0.8%) and *S. haematobium,* hookworm and *Ascaris lumbricoides* (0.2%) were reported. These findings revealed a low prevalence of coinfections, which was similar to the results observed in another study done in Kingwede, Kwale County [19].

Anaemia represents a global public health problem among WRA, pregnant women and children in parasitic endemic areas [20]. The findings of this study indicate a low (16.5%) prevalence of anaemia among WRA in areas of coastal Kenya where *P. falciparum*, *S. haematobium* and STHs are endemic [21]. We evaluated the potential infectious causes of anaemia in WRA in the region and observed that *P. falciparum*, hookworm, *A. lumbricoides and T. trichiura* had no significant correlation with anaemia. These results disagree with most studies, which have shown that there is a positive correlation between parasitic infections and anaemia [22]. Several studies have demonstrated that single infections with either *P. falciparum,* hookworm *and T. trichiura* could result in the reduction of HB [23]. In a study done in the Meheza District in Tanzania, Billy Ngasala and others demonstrated that there was a significant association between malaria or malaria–STHs polyparasitism and anaemia [23]. However, the period of infection, parasitic species and load, physiological iron requirement, body iron stores and dietary intake and absorption may affect the possibility of one getting anaemia [24]. This could explain why the study findings did not support the correlation between parasitic infections and anaemia.

Polyparasitic infections cause more HB reduction compared to single parasitic infections [25]. In this study, the prevalence of anaemia among polyparasitic infected cases was 0.4%. The most common polyparasitic infections recorded and known to cause anaemia were those of *P. falciparum* and *S. haematobium,* and *S. haematobium* and hookworm. These associations and their resultant effects on HB have been highlighted to be common in parasitic endemic areas [23].

This study further determined the correlation between parasitic coinfections and different types of anaemia based on MCV. This is because in resource‐limited settings, clinicians lack testing devices to determine the exact type of anaemia; hence, are left to guess on the corresponding medication, which results in poor treatment response [26]. *P. falciparum* is significantly associated with haemolytic anaemia where microcytosis and hypochromia may be present [27]. Hookworm and *A. lumbricoides*, on the other hand, cause iron deficiency that culminates in microcytic hypochromic anaemia [28]. *S. haematobium* was associated with reduced MCV, while *T. trichiura* was associated with an increase in MCV [29]. In this study, 37% of the infected WRA suffered microcytic anaemia, while 63% had normocytic anaemia. All the 9 (1.8%) polyparasitic cases were positive for microcytic anaemia. These findings demonstrate that microcytic anaemia is common among parasitically coinfected individuals, which concurs with the findings of a study done in 2015 by Cojulun et al. [25]. It is, however, good to note that the results of our study were not conclusive, as the prevalence of coinfection was only 1.8%.

A few recent studies done to explore the morbidity implications of polyparasitic infection thought to elicit some biologic interactions between the invading parasites and the host’s immune system have been done on man [29]. Synergism is one of the possible forms of interaction that exist. This was observed in *Trichinella spiralis* and *Heligmosomoides polygyrus* coinfected mice, where the adverse health effect associated with multiple parasitic species infections was greater than the sum of adverse effects caused by a single parasite species infection [30]. However, there is scanty knowledge on the existence of synergism between murine systems and human populations.

In the current study, the synergistic effect was established by comparing the net effect on mean HB of three polyparasitic infections, namely *P. falciparum* and *S. haematobium, S. haematobium* and hookworm *and S. haematobium,* hookworm and *A. lumbricoides*, and the effect of monoparasitic infections. This study shows that polyparasitic infections had no effect on HB, as the mean HB of all the affected cases was within the acceptable HB range of between 11.0 g/dL and 16.5 g/dL, and thus no synergistic effects for the above polyparasitic interactions and HB were demonstrated. These results disagree with previous studies done in Kwale, Kenya [16]. These results may have failed to clearly demonstrate the possibility of synergism due to the low prevalence of polyparasitic infections that might have resulted from the employed Kato–Katz technique, which is less sensitive in low‐intensity areas such as the study area. A more sensitive method like the FLOTAC technique is recommended for future periodic monitoring of hookworm [31]. Secondly, the use of single urine and stool samples obtained from the participants. This might have led to the low levels of *S. haematobium* and STHs detected. Collection of serial or multiple urine and stool samples always enhances the possibility of finding parasites in the infected person [32]. Lastly, the longer time taken to transport the samples from the field to the designated analysis laboratory might have affected the hookworm reading. It is therefore recommended that STHs, particularly hookworm stool samples, should be analysed immediately after collection.

The study revealed that the burden of polyparasitic infection among WRA was relatively low, with a relatively high prevalence of single infections of *S. haematobium* and *P. falciparum* infections. The predominant and significant parasitic interactions were those of *P. falciparum* and *Schistosoma haematobium*. Both single and polyparasitic interactions recorded were not associated with a high prevalence of anaemia. However, 37% and 63% of the infected WRA had microcytic and normocytic anaemia, respectively.

## Funding

This work was funded by the Kenya National Research Fund (NRF) number NRF/1/MMC/040.

## Disclosure

The funding body had no role in the design of the study, sample collection, analysis and interpretation of the data, and writing and publication of the manuscript.

## Conflicts of Interest

The authors declare no conflicts of interest.

## Data Availability

Data are available upon reasonable request from the corresponding author.
